# Xenobiotic metabolizing enzyme gene polymorphisms predict response to lung volume reduction surgery

**DOI:** 10.1186/1465-9921-8-59

**Published:** 2007-08-08

**Authors:** Craig P Hersh, Dawn L DeMeo, John J Reilly, Edwin K Silverman

**Affiliations:** 1 Channing Laboratory, Department of Medicine, Brigham and Women's Hospital, Boston, MA, USA; 2 Division of Pulmonary and Critical Care Medicine, Brigham and Women's Hospital, Boston, MA, USA; 3 Harvard Medical School, Boston, MA, USA

## Abstract

**Background:**

In the National Emphysema Treatment Trial (NETT), marked variability in response to lung volume reduction surgery (LVRS) was observed. We sought to identify genetic differences which may explain some of this variability.

**Methods:**

In 203 subjects from the NETT Genetics Ancillary Study, four outcome measures were used to define response to LVRS at six months: modified BODE index, post-bronchodilator FEV_1_, maximum work achieved on a cardiopulmonary exercise test, and University of California, San Diego shortness of breath questionnaire. Sixty-four single nucleotide polymorphisms (SNPs) were genotyped in five genes previously shown to be associated with chronic obstructive pulmonary disease susceptibility, exercise capacity, or emphysema distribution.

**Results:**

A SNP upstream from glutathione S-transferase pi (*GSTP1*; p = 0.003) and a coding SNP in microsomal epoxide hydrolase (*EPHX1*; p = 0.02) were each associated with change in BODE score. These effects appeared to be strongest in patients in the non-upper lobe predominant, low exercise subgroup. A promoter SNP in *EPHX1 *was associated with change in BODE score (p = 0.008), with the strongest effects in patients with upper lobe predominant emphysema and low exercise capacity. One additional SNP in *GSTP1 *and three additional SNPs in *EPHX1 *were associated (p < 0.05) with additional LVRS outcomes. None of these SNP effects were seen in 166 patients randomized to medical therapy.

**Conclusion:**

Genetic variants in *GSTP1 *and *EPHX1*, two genes encoding xenobiotic metabolizing enzymes, were predictive of response to LVRS. These polymorphisms may identify patients most likely to benefit from LVRS.

## Background

The National Emphysema Treatment Trial, a multicenter randomized trial of lung volume reduction surgery (LVRS) versus medical management for emphysema, found that on average, LVRS led to improved functional status, but not increased survival in patients with emphysema and severe chronic airflow obstruction [1]. However, substantial variability in response to LVRS was observed. Based on pulmonary function testing and emphysema distribution on chest computed tomography (CT), a patient population with a high risk of death was identified [2]. Among non-high risk patients, baseline exercise capacity and emphysema distribution on chest CT scans were used to define subgroups with greater or lesser chances of improvement post-LVRS. Yet these clinical subgroups did not fully account for the variable response to LVRS among NETT participants.

We hypothesized that genetic differences may explain some of this variability in response to LVRS. To test this hypothesis, we studied participants in the NETT Genetics Ancillary Study. We examined the association between LVRS outcomes and variants in five genes previously shown to be associated with chronic obstructive pulmonary disease (COPD) susceptibility, exercise capacity, or emphysema distribution on chest CT [3-7]: glutathione S-transferase pi (*GSTP1*), microsomal epoxide hydrolase (*EPHX1*), transforming growth factor beta-1 (*TGFB1*), serpin peptidase inhibitor E2 (*SERPINE2*) and surfactant, pulmonary-associated protein B (*SFTPB*). Though not a "pharmacogenetic" study in the classic sense of the term – since the intervention studied is a surgical procedure and not a pharmacological agent – the present study is the first to examine genetic associations for response to a specific therapy for COPD.

## Methods

### Study Subjects

Study enrollment and phenotype measurements in NETT have been reported [1,8]. Subjects enrolled in NETT had severe airflow obstruction (FEV_1 _≤ 45% predicted), hyperinflation (total lung capacity ≥ 100% predicted), and bilateral emphysema on high-resolution chest CT. Subjects were excluded if they had major comorbid illnesses, including significant cardiovascular disease, interstitial lung disease, or malignancy. Maximal exercise capacity was determined by incremental cycle ergometry [1]. The University of California, San Diego, shortness of breath questionnaire (UCSD SOBQ) was used to quantify dyspnea, with higher scores indicating more severe dyspnea [1,9]. Spirometry was performed according to ATS standards [1,10]. The BODE (Body mass index, airflow Obstruction, Dyspnea, Exercise capacity) index is a 10-point composite score in which higher scores predicted poorer emphysema outcomes [11]. We modified the BODE index to include the UCSD SOBQ as the dyspnea instrument instead of the Medical Research Council dyspnea scale, which was not available in NETT [6,12]. The other components of the BODE index included body mass index, post-bronchodilator FEV_1 _(% predicted), and 6-minute walk test distance [11].

In the NETT Genetics Ancillary Study, participants were re-contacted by the sixteen participating NETT Centers. After written informed consent, subjects provided a blood sample for DNA extraction. To limit genetic heterogeneity, the analysis was limited to non-Hispanic white participants without severe α1-antitrypsin deficiency; a total of 203 LVRS patients and 166 medically treated patients were included. The NETT Genetics Ancillary Study was approved by the institutional review boards at participating NETT centers.

### Genotyping

Single nucleotide polymorphisms (SNPs) were selected in five genes: *GSTP1*, *EPHX1*, *TGFB1*, *SERPINE2 *and *SFTPB*. We used genotype data from European-Americans (CEU) in the International HapMap project [13] and in the SeattleSNPs database [14] to select a set of linkage disequilibrium (LD)-tagging SNPs for each gene. Pairwise LD-tagging was implemented in Tagger [15], with a minimum minor allele frequency of 0.1 and r^2 ^threshold of 0.9. Specific SNPs previously associated with COPD or related traits were also included.

The 64 SNPs were genotyped on one of three platforms (see Additional file 1): allele specific hybridization (Illumina Golden Gate assay, San Diego, CA), the 5' to 3' exonuclease assay (TaqMan, Applied Biosystems, Foster City, CA) or with unlabeled minisequencing reactions and mass spectrometry (Sequenom, San Diego, CA).

### Statistical Analysis

Four outcome measurements were analyzed: modified BODE index [11,12], post-bronchodilator FEV_1 _(liters), maximum work achieved on a cardiopulmonary exercise test, and the UCSD SOBQ score [9]. We considered outcome measurements at six months following randomization, in order to allow for recovery from surgery, but to precede the loss of benefit from LVRS that occurs over time [16]. LVRS response was defined as the difference between this measurement and the baseline, recorded following pulmonary rehabilitation, but prior to randomization.

Genotype-phenotype correlations were assessed by linear regression, with adjustment for age, sex, and pack-years of smoking, assuming additive genetic models by testing for a linear trend across 0, 1, and 2 copies of the minor allele. Models for FEV_1 _and maximum work were additionally adjusted for height. As a secondary analysis, stratified analyses were performed in the four subgroups defined in NETT [1]. Similar models were performed in subjects in the NETT Genetics Ancillary Study who had been randomized to medical therapy. Analyses were conducted using SAS version 9.1 (SAS Institute, Cary, NC) or R [17]. LD was calculated using Haploview [18]. Statistical power was estimated using Quanto [19], assuming additive genetic models, with a two-sided α = 0.05. Putative transcription factor binding sites were identified with MAPPER [20].

## Results

### Study Subjects

**Figure 1:**
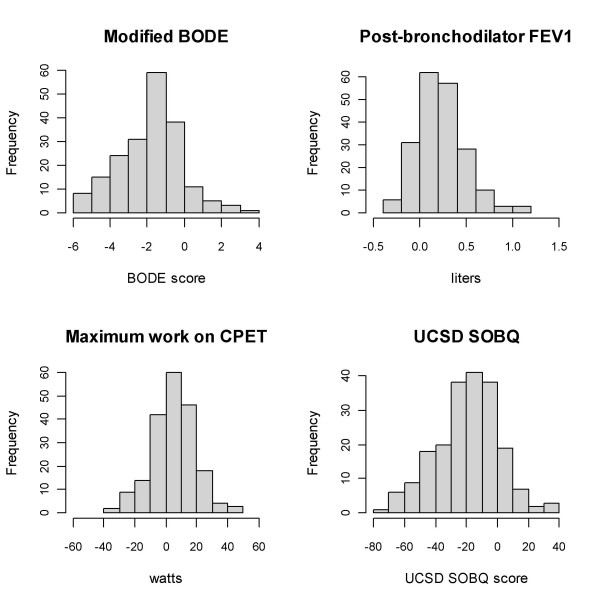
Frequency distributions of changes in outcomes at six months in 203 lung volume reduction surgery patients in the NETT Genetics Ancillary Study. BODE = Body mass index, airflow Obstruction Dyspnea Exercise tolerance; FEV_1_= forced expiratory volume in 1 second; CPET = cardiopulmonary exercise test; UCSD SOBQ = University of California, San Diego shortness of breath questionnaire

Characteristics of the 203 non-Hispanic white participants in the NETT Genetics Ancillary Study who underwent LVRS are shown in Table 1. These subjects resembled the full cohort of 608 patients randomized to LVRS in NETT [1]. Outcomes at six months are also shown in Table 1. On average, participants showed improvement post-LVRS, with increases in FEV_1 _and exercise capacity and decreases in BODE score and dyspnea. However, Figure 1 demonstrates the variability in response to LVRS among study participants.

**Table 1:** Characteristics of NETT Genetics Ancillary Study subjects who underwent lung volume reduction surgery (LVRS). N = 203 unless otherwise noted

*Characteristic*	*Mean (SD) or N(%)*
*Baseline (pre-randomization)*	
Age, years	67.5 (6.2)
Male sex	123 (60.6%)
Pack-years of smoking	65.2 (29.6)
Upper lobe predominant emphysema	143 (70.4%)
Low exercise capacity	82 (40.4%)
Modified BODE score	4.7 (1.6)
Post-bronchodilator FEV_1_, liters	0.80 (0.26)
Post-bronchodilator FEV_1_, % predicted	28.2 (7.5)
Maximum work achieved on CPET, watts	41.8 (21.9)
UCSD shortness of breath questionnaire	58.7 (17.5)
	
*Change at 6 months post-LVRS*	
Modified BODE score (N = 195)	-1.3 (1.7)
Post-bronchodilator FEV_1_, liters (N = 200)	0.23 (0.26)
Post-bronchodilator FEV_1_, % predicted (N = 200)	8.9 (9.8)
Maximum work achieved on CPET, watts (N = 198)	6.4 (14.1)
UCSD shortness of breath questionnaire (N = 202)	-18.7 (20.7)

BODE = Body mass index, airflow Obstruction, Dyspnea, Exercise tolerance;

CPET = cardiopulmonary exercise test; FEV_1 _= forced expiratory volume in 1 second;

UCSD = University of California, San Diego

### LVRS Response

The genotype frequencies of all 64 SNPs conformed to the expectation under Hardy-Weinberg Equilibrium (at a threshold of p < 0.01), except for one SNP in *GSTP1*, rs1799811; this SNP was removed from subsequent analyses. The results of the association analyses for the remaining 63 SNPs are highlighted in Table 2. A SNP 5' to *GSTP1 *and a promoter and a coding SNP in *EPHX1 *were significantly (p < 0.05) associated with change in BODE score; the promoter SNP in *EPHX1 *was also associated with change in UCSD SOBQ score. A SNP 3' to *GSTP1 *was significantly associated with changes in post-bronchodilator FEV_1 _and maximum work. An intronic SNP in *EPHX1 *was associated with FEV_1 _change. Two additional SNPs in *EPHX1 *(rs1051741 and rs2292558), in strong LD with each other (r^2 ^= 0.97), were associated with change in maximum work. The two SNPs in *GSTP1 *and the promoter SNP in *EPHX1 *remained significant when a p-value <0.01 was used to define significance, as an adjustment for the five genes tested.

**Table 2:** Genetic associations with lung volume reduction surgery response at 6 months. Associations with p-values < 0.1 are shown. All analyses are adjusted for age, sex, and pack-years of smoking. Analyses of FEV_1 _and maximum work are also adjusted for height

Gene (total SNPs)	SNP	Minor allele frequency	BODE score	Post-BD FEV_1_	Maximum work	USCD SOBQ score
*GSTP1 *(7)	rs612020 5' genomic	0.083	0.003			
	rs11227884 3' genomic	0.073		0.003	0.02	
						
*SFTPB *(5)	--*					
						
*SERPINE2 *(22)	rs6436449 intron	0.17	0.07			0.09
						
*EPHX1 *(19)	rs3753658 promoter	0.10	0.008			0.02
	rs1877724 intron	0.26		0.04		
	rs2234922 His139Arg	0.19	0.02		0.05	
	rs1051741 exon, synon.	0.11	0.09		0.01	
	rs360063 3' genomic	0.38	0.07	0.05		
	rs2292558 3' genomic	0.11	0.09		0.03	
	rs1009668 3' genomic	0.10		0.08		
						
*TGFB1 *(11)	rs2241712 promoter	0.32	0.07			
	rs8110090 intron	0.05		0.09		
	rs8179181 intron	0.26			0.06	
	rs12981053 3' genomic	0.15		0.06		
	rs12980942 3' genomic	0.15		0.05		

SNP = single nucleotide polymorphism; BD = bronchodilator; FEV_1 _= forced expiratory volume in 1 second; BODE = Body mass index, airflow Obstruction, Dyspnea, Exercise tolerance; UCSD SOBQ = University of California, San Diego shortness of breath questionnaire

*No SNPs with p-value < 0.1

One SNP in *SERPINE2 *and a total of five SNPs in *TGFB1 *showed trends for association with one or more LVRS response phenotypes, but none were significant at p < 0.05. None of the SNPs tested in *SFTPB *were significantly associated.

### Subgroup Analyses

The significant genotype-phenotype associations for SNPs in *GSTP1 *and *EPHX1 *were further evaluated in four clinically-defined subgroups of patients in NETT [1], based on emphysema distribution and baseline exercise capacity. Emphysema distribution was categorized as upper lobe predominant or non-upper lobe predominant, based on the radiologist's interpretation of the chest CT scan. Low baseline exercise capacity was defined by sex-specific thresholds of maximum work achieved on cycle ergometry (≤ 40 watts for men or ≤ 25 watts for women). Distribution of subjects in each subgroup is as follows: upper lobe predominant, low exercise capacity 59 (29.1%); upper lobe predominant, high exercise capacity 84 (41.4%); non-upper lobe predominant, low exercise capacity 23 (11.3%); non-upper lobe predominant, high exercise capacity 37 (18.2%). The fourteen patients who would be defined as high-risk [2] were not excluded from the subgroup analysis, due to the already limited number of subjects in the subgroups.

**Figure 2:**
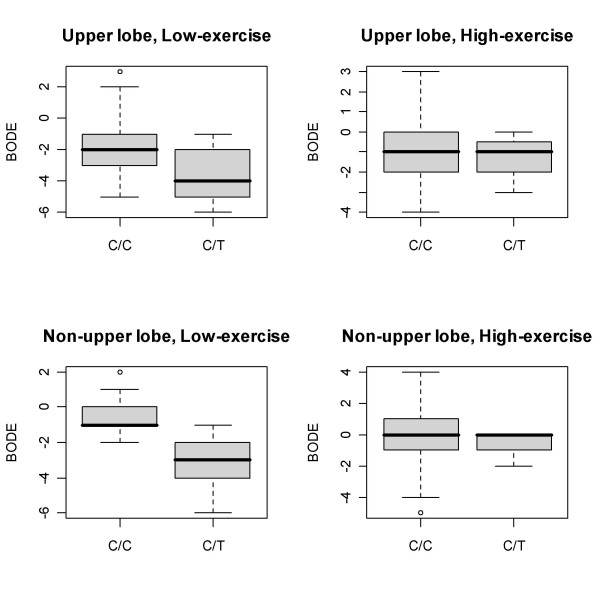
Effect of *GSTP1* rs612020 polymorphism in patient subgroups defined by emphysema distribution and baseline exercise capacity. Six month change in BODE score is shown. The grey box represents the interquartile range, and the black line marks the median. One individual with T/T genotype has been removed for clarity of presentation.

Because of the small sample sizes, only SNPs that were significantly associated (p < 0.05) with a specific phenotype in all subjects were examined in an exploratory subgroup analysis. Table 3 shows the subgroup analysis for SNPs in *GSTP1 *that were significantly associated with at least one trait in all subjects. The minor allele of rs612020, located 5' to the *GSTP1 *transcript, was associated with a reduction in BODE score (signifying clinical improvement) in all subjects. This SNP was associated with greater improvement in patients with low exercise capacity, with both upper lobe predominant and non-upper lobe predominant emphysema (Figure 2). In the non-upper predominant, low exercise capacity subgroup, the effect of the SNP was more than twice that in all subjects; the p-value was the same, despite the marked reduction in sample size. In all subjects, the minor allele at rs11227884, 3' to the *GSTP1 *gene, was associated with improvement in FEV_1 _and maximum work. The effect of the SNP on maximum work was stronger in the upper lobe predominant, low exercise capacity subgroup, though that association did not reach statistical significance.

**Table 3:** Analysis of SNPs in *GSTP1 *in all subjects (significant at p < 0.05) and in 4 subgroups defined by NETT based on upper lobe predominant emphysema on chest CT (upper lobe predominant vs. non-upper lobe predominant) and baseline exercise capacity (low vs. high). Subgroups with p-value <0.1 are shown

SNP	LVRS Response Phenotype	All subjects (n = 203)		Upper lobe, low exercise (n = 59)		Upper lobe, high exercise (n = 84)		Non-upper lobe, low exercise (n = 23)		Non-upper lobe, high exercise (n = 37)	
		
		β	p	β	p	β	p	β	p	β	p
rs612020	BODE	-1.0	0.003	-1.5	0.03			-2.2	0.003		
rs11227884	Post-BD FEV_1_, liters	0.15	0.003								
	Max work, watts	6.2	0.02	8.5	0.06						

SNP = single nucleotide polymorphism; LVRS = lung volume reduction surgery; BODE = Body mass index, airflow Obstruction, Dyspnea, Exercise tolerance; BD = bronchodilator; FEV_1 _= forced expiratory volume in 1 second

**Figure 3:**
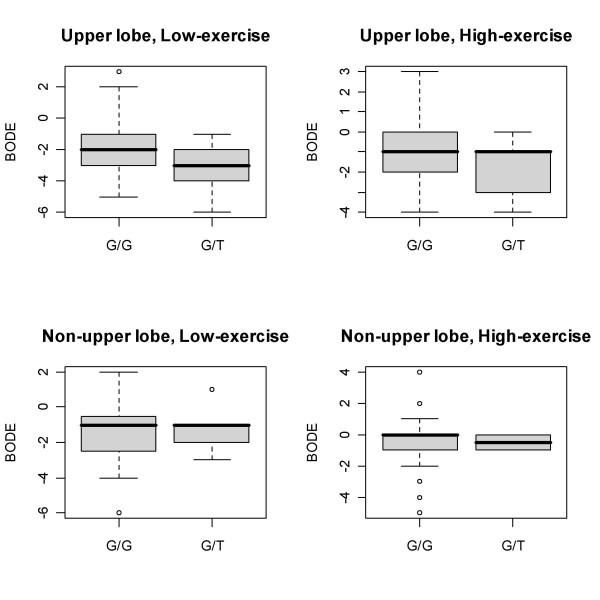
Effect of *EPHX1 *rs3753658 promoter polymorphism in patient subgroups defined by emphysema distribution and baseline exercise capacity. Six month change in BODE score is shown. The grey box represents the interquartile range, and the black line marks the median. Three individuals with T/T genotype have been removed for clarity of presentation.

A similar stratified analysis for SNPs in *EPHX1 *is detailed in Table 4. A promoter SNP in *EPHX1*, rs375658, was associated with decreased BODE index and decreased USCD SOBQ score, both representing clinical improvement, in all subjects. These effects were stronger in the upper lobe predominant, low exercise capacity subgroup (Figure 3). An intronic SNP in *EPHX1*, rs1877724, was associated with a slight worsening in FEV_1_, again with stronger effects in upper lobe predominant, low exercise capacity patients. The His139Arg coding variant (rs2234922) was associated with worsening BODE score and a decrease in exercise capacity, with the effects on BODE score stronger in the non-upper lobe predominant, low exercise capacity subgroup. Two additional SNPs in *EPHX1 *showed an association with worsening exercise capacity. The effects were stronger in both non-upper lobe predominant subgroups, though not statistically significant.

**Table 4:** Analysis of SNPs in *EPHX1 *in all lung volume reduction surgery subjects (significant at p < 0.05) and in 4 subgroups defined by NETT based on upper lobe predominant emphysema on chest CT (upper lobe predominant vs. non-upper lobe predominant) and baseline exercise capacity (low vs. high). Subgroups with p-value <0.1 are shown

SNP	LVRS Response Phenotype	All subjects (n = 203)		Upper lobe, low exercise (n = 59)		Upper lobe, high exercise (n = 84)		Non-upper lobe, low exercise (n = 23)		Non-upper lobe, high exercise (n = 37)	
		
		β	p	β	p	β	p	β	p	β	p
rs3753658	BODE	-0.8	0.008	-1.3	0.01						
	UCSD SOBQ	-7.7	0.02	-11.7	0.05						
rs1877724	Post-BD FEV_1_, liters	-0.06	0.04	-0.13	0.01						
rs2234922	BODE	0.5	0.02					1.6	0.02		
	Max work, watts	-3.7	0.05								
rs1051741	Max work, watts	-5.9	0.01					-8.4	0.09	-10.2	0.09
rs2292558	Max work, watts	-5.1	0.03					-8.4	0.09	-10.2	0.09

SNP = single nucleotide polymorphism; LVRS = lung volume reduction surgery; BODE = Body mass index, airflow Obstruction, Dyspnea, Exercise tolerance; BD = bronchodilator; FEV_1 _= forced expiratory volume in 1 second; UCSD SOBQ = University of California, San Diego shortness of breath questionnaire (higher scores indicate more severe dyspnea)

### Post-Operative Complications

To explore the possibility that the significant SNPs were affecting LVRS response through effects on post-operative complications, we re-analyzed the two significant (p < 0.05) SNPs in *GSTP1 *and five significant SNPs in *EPHX1 *after excluding fourteen patients with a post-LVRS hospital length of stay greater than thirty days. The effect estimates and p-values for the two *GSTP1 *SNPs were not substantially changed. In *EPHX1*, the promoter SNP (rs3753658) was associated with greater improvement (BODE β = -1.0, p = 0.003; UCSD SOBQ β = -10.5, p = 0.004). The His139Arg SNP was less detrimental (BODE β = 0.4, p = 0.1; maximum work β = -2.9, p = 0.1), though this effect was not statistically significant. The effects of the other three SNPs in *EPHX1 *were unchanged.

### Medical Arm

In order to ensure that the SNP effects seen in the LVRS patients were not merely reflective of the natural history of severe emphysema, we examined 166 patients from the NETT Genetics Ancillary Study who had been randomized to medical therapy. The two SNPs in *GSTP1 *and five SNPs in *EPHX1 *that were significant in all LVRS patients were tested for association. None of these genotype-phenotype associations were significant in subjects from the medical arm. Despite the smaller sample size in the medical arm, power was reasonable to detect significant genetic associations. For *GSTP1 *SNP rs612020, the medical arm had 93% power to detect a similar effect on BODE score as was seen in the LVRS patients. For *EPHX1 *SNPs rs3753658 and rs2234922 (His139Arg), power was 97% and 78%, respectively, for the analyses of BODE in the medical arm.

## Discussion

In participants from the NETT Genetics Ancillary Study, we tested associations between variants in five candidate genes and four measures of response to LVRS, finding significant associations for SNPs in two genes, *GSTP1 *and *EPHX1*. The effects of a SNP upstream from *GSTP1 *and a coding SNP in *EPHX1 *were strongest in the clinically defined subgroup of patients with non-upper lobe predominant emphysema and low baseline exercise tolerance. Additional SNPs in these two genes, including a promoter SNP in *EPHX1*, appeared to have stronger effects in patients with upper lobe predominant emphysema and low baseline exercise tolerance.

Analysis of the NETT data has demonstrated that non-high risk patients in the upper lobe predominant, low baseline exercise capacity subgroup are most likely to benefit from LVRS, with a survival advantage compared to medical therapy [1]. Based on these results and previous studies of LVRS [21], LVRS is widely accepted for patients with severe airflow obstruction due to upper lobe predominant emphysema. Our findings in the upper lobe predominant, low exercise capacity subgroup may distinguish a subset of these patients most likely to respond to surgery. However, the role of LVRS for non-upper lobe predominant emphysema is much less clear [16]. NETT found no survival improvement from LVRS in the non-upper lobe predominant, low baseline exercise capacity subgroup, but did show the potential for symptomatic benefit in these patients [1]. The genetic associations in this subgroup may possibly identify patients with non-upper lobe predominant emphysema who have the potential to benefit from LVRS. However, the number of patients included in this subgroup was small.

In contrast to traditional pharmacogenetic studies of drugs and their metabolizing enzymes, the potential effect of SNPs in *GSTP1 *and *EPHX1*, two genes encoding xenobiotic metabolizing enzymes, on the response to LVRS is not obvious. Variants in these genes may influence an individual's response to the inflammation produced by surgery or to the oxidative stress resulting from single lung ventilation during lung resection [22]. Alternatively, these genetic variants may be identifying patients with different subtypes of emphysema, beyond the subgroups defined by radiographic distribution and baseline exercise capacity. The fact that we could not replicate these associations in patients randomized to medical therapy demonstrates that the effects of these SNPs are not explained by genetic influences on the natural history of emphysema with severe airflow obstruction. The effects of variants in *EPHX1 *may be at least partially mediated through effects on the post-operative course, including complications, evidenced by the change in effect estimates in the analyses excluding patients with post-LVRS hospital stays greater than thirty days. It is unlikely that the associated SNPs are exerting their effects through comorbid illnesses, since the number of major comorbidities in NETT subjects was low due to the study exclusion criteria [23].

One must also consider the potential effects of the specific SNPs that we have determined to be significantly associated with LVRS outcome. A coding variant in *GSTP1 *(Ile105Val) has been associated with COPD and related traits in several studies [24,25], but the results have not been consistently replicated [5,26]. The SNP with the strongest association in our study, rs612020, is located upstream from the transcription start site of the *GSTP1 *gene. The functional effect of this particular SNP is not clear, yet it is in complete LD (in European-Americans from the HapMap project) with another upstream SNP, rs7927381 (which was not genotyped in our study), which may alter a putative CCAAT/enhancer-binding protein (CEBP) site. The transcription factor CEBP-γ may be an important regulator of *GSTP1 *expression in human bronchial epithelial cells [27].

In *EPHX1*, rs3753658 is in the promoter region, 290 bp upstream from the transcription start site. The SNP is in complete LD with another promoter SNP (rs3753660, not genotyped in our study) [28], which may affect a binding site for peroxisome proliferator-activated receptor-γ, a modulator of airway inflammation in COPD [29]. SNP rs2234922 is located in exon 4 and leads to an amino acid change (His139Arg). Enzymes carrying this variation may have increased activity [30]; this variant has been termed the "fast" allele. Several studies have reported association between another coding variant (Tyr113His, "slow" allele) and COPD [31,32]. As with *GSTP1*, this finding has not been consistently replicated. We have previously reported a protective effect of the His139Arg variant on COPD risk, comparing patients from NETT with control subjects [5]; however, this association was not found in a family-based study of COPD.

The published studies of *GSTP1 *and *EPHX1 *above have largely examined associations with COPD susceptibility. The present study is the first association analysis examining genetic influences on the response to a specific therapy for COPD or emphysema. In a study of outcomes from thoracic surgery, Shaw and colleagues genotyped six polymorphisms in five genes, finding associations for SNPs in tumor necrosis factor (TNF) and interleukin-6 (IL6) with the risk of complications in 155 patients undergoing lung resections for cancer [33]. On average, their patients had relatively preserved baseline pulmonary function. In addition, multiple studies have examined genetic and genomic factors influencing outcomes from cardiac surgery [34].

Our study has several limitations. In NETT, DNA samples were collected at various times following enrollment, and not prior to randomization. Because subjects were recruited into the NETT Genetics Ancillary Study after enrollment into NETT, we could not examine whether genetic variants influenced survival post-LVRS, since patients who died soon after enrollment (e.g. peri-operative deaths) would not be included in the study.

In our analyses of four phenotypes and five genes, including multiple SNPs in those genes, it is possible that the positive results represent spurious associations due to the multiple tests performed. Using a more stringent p-value of 0.01, only three of the genotype-phenotype associations in our study (two SNPs in *GSTP1 *and one in *EPHX1*) remained significant. In the complex trait genetics literature, there is no clear consensus regarding the optimal statistical methodology to control for multiple testing [35]. Increasingly, replication of the findings in an independent population has emerged as the standard for confirming a true genetic association [36]. A limitation of our study is the lack of a suitable replication population. Other clinical trials of LVRS [21] would likely be underpowered for an adequate replication study, even if DNA were collected on all subjects in these studies. For example, in the combined analysis of the Canadian Lung Volume Reduction Study and the Overholt-Blue Cross Emphysema Surgery Trial, one of the largest LVRS trials outside of NETT, only 58 patients were randomized to surgery [37]. For a replication study, ideally one targets a sample size at least as large as in the original study [38].

## Conclusion

In the NETT Genetics Ancillary Study, we were able to identify variants in two genes, *GSTP1 *and *EPHX1*, which may predict outcome from LVRS, even when accounting for clinically-defined subgroups based on radiographic emphysema distribution and baseline exercise capacity. This represents the first genetic association for response to a specific therapy for COPD. Given that an adequate clinical trial population for replication is unlikely to become available, alternative methodologies must be employed to validate our findings and to confirm their eventual clinical relevance.

## Abbreviations

BODE = Body mass index, airflow Obstruction, Dyspnea, Exercise tolerance

COPD = chronic obstructive pulmonary disease

FEV_1 _= forced expiratory volume in 1 second

LD = linkage disequilibrium

LVRS = lung volume reduction surgery

NETT = National Emphysema Treatment Trial

SNP = single nucleotide polymorphism

UCSD SOBQ = University of California, San Diego shortness of breath questionnaire

## Competing interests

Dr. Silverman has received honoraria, consultant fees, and research grants from GlaxoSmithKline for COPD genetics studies and honoraria from Wyeth, Bayer, and Astra-Zeneca for lectures on COPD genetics. None of the other authors report any relevant competing interests.

## Authors' contributions

CPH designed the analysis, participated in data collection, performed the analyses and interpretation of results, and drafted the manuscript. DLD participated in the conceptualization of the analysis, data collection, and revision of the manuscript. JJR participated in the conceptualization of the analysis, subject recruitment, and revision of the manuscript. EKS participated in the design of the analysis, subject recruitment, data collection, interpretation of the results, and revision of the manuscript. All authors have read and approved the final manuscript.

## Supplementary Material

Additional file 1 Single nucleotide polymorphisms (SNP) and genotyping platforms. SNPs genotyped in the NETT Genetics Ancillary Study subjects. Map locations are based on the May 2004 human genome reference sequence (National Center for Biotechnology Information [NCBI] Build 35). Click here for file

## References

[B1] Fishman A, Martinez F, Naunheim K, Piantadosi S, Wise R, Ries A, Weinmann G, Wood DE (2003). A randomized trial comparing lung-volume-reduction surgery with medical therapy for severe emphysema. N Engl J Med.

[B2] National Emphysema Treatment Trial Research Group (2001). Patients at high risk of death after lung-volume-reduction surgery. N Engl J Med.

[B3] Celedon JC, Lange C, Raby BA, Litonjua AA, Palmer LJ, DeMeo DL, Reilly JJ, Kwiatkowski DJ, Chapman HA, Laird N, Sylvia JS, Hernandez M, Speizer FE, Weiss ST, Silverman EK (2004). The transforming growth factor-{beta}1 (TGFB1) gene is associated with chronic obstructive pulmonary disease (COPD). Hum Mol Genet.

[B4] Demeo DL, Mariani TJ, Lange C, Srisuma S, Litonjua AA, Celedon JC, Lake SL, Reilly JJ, Chapman HA, Mecham BH, Haley KJ, Sylvia JS, Sparrow D, Spira AE, Beane J, Pinto-Plata V, Speizer FE, Shapiro SD, Weiss ST, Silverman EK (2006). The SERPINE2 Gene Is Associated with Chronic Obstructive Pulmonary Disease. Am J Hum Genet.

[B5] Hersh CP, Demeo DL, Lange C, Litonjua AA, Reilly JJ, Kwiatkowski D, Laird N, Sylvia JS, Sparrow D, Speizer FE, Weiss ST, Silverman EK (2005). Attempted replication of reported chronic obstructive pulmonary disease candidate gene associations. Am J Respir Cell Mol Biol.

[B6] Hersh CP, Demeo DL, Lazarus R, Celedon JC, Raby BA, Benditt JO, Criner G, Make B, Martinez FJ, Scanlon PD, Sciurba FC, Utz JP, Reilly JJ, Silverman EK (2006). Genetic association analysis of functional impairment in chronic obstructive pulmonary disease. Am J Respir Crit Care Med.

[B7] Demeo DL, Hersh CP, Hoffman EA, Litonjua AA, Lazarus R, Sparrow D, Benditt JO, Criner G, Make B, Martinez FJ, Scanlon PD, Sciurba FC, Utz JP, Reilly JJ, Silverman EK (2007). Genetic determinants of emphysema distribution in the national emphysema treatment trial. Am J Respir Crit Care Med.

[B8] The National Emphysema Treatment Trial Research Group (1999). Rationale and design of The National Emphysema Treatment Trial: a prospective randomized trial of lung volume reduction surgery.. Chest.

[B9] Eakin EG, Resnikoff PM, Prewitt LM, Ries AL, Kaplan RM (1998). Validation of a new dyspnea measure: the UCSD Shortness of Breath Questionnaire. University of California, San Diego. Chest.

[B10] American Thoracic Society (1995). Standardization of Spirometry, 1994 Update.. Am J Respir Crit Care Med.

[B11] Celli BR, Cote CG, Marin JM, Casanova C, Montes de Oca M, Mendez RA, Pinto Plata V, Cabral HJ (2004). The body-mass index, airflow obstruction, dyspnea, and exercise capacity index in chronic obstructive pulmonary disease. N Engl J Med.

[B12] Martinez FJ, Foster G, Curtis JL, Criner G, Weinmann G, Fishman A, DeCamp MM, Benditt J, Sciurba F, Make B, Mohsenifar Z, Diaz P, Hoffman E, Wise R (2006). Predictors of mortality in patients with emphysema and severe airflow obstruction. Am J Respir Crit Care Med.

[B13] (2003). The International HapMap Project. Nature.

[B14] SeattleSNPs. http://pga.gs.washington.edu/.

[B15] de Bakker PI, Yelensky R, Pe'er I, Gabriel SB, Daly MJ, Altshuler D (2005). Efficiency and power in genetic association studies. Nat Genet.

[B16] Naunheim KS, Wood DE, Mohsenifar Z, Sternberg AL, Criner GJ, DeCamp MM, Deschamps CC, Martinez FJ, Sciurba FC, Tonascia J, Fishman AP (2006). Long-term follow-up of patients receiving lung-volume-reduction surgery versus medical therapy for severe emphysema by the National Emphysema Treatment Trial Research Group. Ann Thorac Surg.

[B17] The R Project for Statistical Computing. http://www.r-project.org/.

[B18] Barrett JC, Fry B, Maller J, Daly MJ (2005). Haploview: analysis and visualization of LD and haplotype maps. Bioinformatics.

[B19] Gauderman WJ (2002). Sample size requirements for matched case-control studies of gene-environment interaction. Stat Med.

[B20] Marinescu VD, Kohane IS, Riva A (2005). MAPPER: a search engine for the computational identification of putative transcription factor binding sites in multiple genomes. BMC Bioinformatics.

[B21] Berger RL, Wood KA, Cabral HJ, Goodnight-White S, Ingenito EP, Gray A, Miller J, Springmeyer SC (2005). Lung volume reduction surgery: a meta-analysis of randomized clinical trials. Treat Respir Med.

[B22] Cheng YJ, Chan KC, Chien CT, Sun WZ, Lin CJ (2006). Oxidative stress during 1-lung ventilation. J Thorac Cardiovasc Surg.

[B23] Fan VS, Ramsey SD, Make BJ, Martinez FJ (2007). Physiologic variables and functional status independently predict COPD hospitalizations and emergency department visits in patients with severe COPD. COPD.

[B24] He JQ, Connett JE, Anthonisen NR, Pare PD, Sandford AJ (2004). Glutathione S-transferase variants and their interaction with smoking on lung function. Am J Respir Crit Care Med.

[B25] Ishii T, Matsuse T, Teramoto S, Matsui H, Miyao M, Hosoi T, Takahashi H, Fukuchi Y, Ouchi Y (1999). Glutathione S-transferase P1 (GSTP1) polymorphism in patients with chronic obstructive pulmonary disease. Thorax.

[B26] Yim JJ, Yoo CG, Lee CT, Kim YW, Han SK, Shim YS (2002). Lack of association between glutathione S-transferase P1 polymorphism and COPD in Koreans. Lung.

[B27] Mullins DN, Crawford EL, Khuder SA, Hernandez DA, Yoon Y, Willey JC (2005). CEBPG transcription factor correlates with antioxidant and DNA repair genes in normal bronchial epithelial cells but not in individuals with bronchogenic carcinoma. BMC Cancer.

[B28] Raaka S, Hassett C, Omiencinski CJ (1998). Human microsomal epoxide hydrolase: 5'-flanking region genetic polymorphisms. Carcinogenesis.

[B29] Huang TH, Razmovski-Naumovski V, Kota BP, Lin DS, Roufogalis BD (2005). The pathophysiological function of peroxisome proliferator-activated receptor-gamma in lung-related diseases. Respir Res.

[B30] Hassett C, Aicher L, Sidhu JS, Omiecinski CJ (1994). Human microsomal epoxide hydrolase: genetic polymorphism and functional expression in vitro of amino acid variants. Hum Mol Genet.

[B31] Sandford AJ, Chagani T, Weir TD, Connett JE, Anthonisen NR, Pare PD (2001). Susceptibility genes for rapid decline of lung function in the lung health study. Am J Respir Crit Care Med.

[B32] Smith CA, Harrison DJ (1997). Association between polymorphism in gene for microsomal epoxide hydrolase and susceptibility to emphysema. Lancet.

[B33] Shaw AD, Vaporciyan AA, Wu X, King TM, Spitz MR, Putnam JB, Dickey BF (2005). Inflammatory gene polymorphisms influence risk of postoperative morbidity after lung resection. Ann Thorac Surg.

[B34] Podgoreanu MV, Schwinn DA (2005). New paradigms in cardiovascular medicine: emerging technologies and practices: perioperative genomics. J Am Coll Cardiol.

[B35] Balding DJ (2006). A tutorial on statistical methods for population association studies. Nat Rev Genet.

[B36] Hirschhorn JN, Altshuler D (2002). Once and again-issues surrounding replication in genetic association studies. J Clin Endocrinol Metab.

[B37] Miller JD, Berger RL, Malthaner RA, Celli BR, Goldsmith CH, Ingenito EP, Higgins D, Bagley P, Cox G, Wright CD (2005). Lung volume reduction surgery vs medical treatment: for patients with advanced emphysema. Chest.

[B38] Colhoun HM, McKeigue PM, Davey Smith G (2003). Problems of reporting genetic associations with complex outcomes. Lancet.

